# Supplementary material for: National climate action can ameliorate, perpetuate, or exacerbate international air pollution inequalities
Source: Nat Commun. 2026 Jan 26;17:1649. doi: 10.1038/s41467-026-68827-0 (PMC12909814; doi:10.1038/s41467-026-68827-0)
Supplement: Supplementary file 1 — Supplementary Information [file 41467_2026_68827_MOESM1_ESM.pdf]

Supplementary Information for

National climate action can ameliorate, perpetuate, or exacerbate international air pollution inequalities

M. Omar Nawaz[1,2,3*] and Daven K. Henze[3]

[1]School of Earth and Environmental Sciences, Cardiff University, Cardiff, Wales, United Kingdom CF10 3AT

[2]Department of Environmental and Occupational Health, George Washington University, Washington, DC, USA 20037

[3]Department of Mechanical Engineering, University of Colorado Boulder, Boulder, CO, USA, 80309

*Correspondence to M. Omar Nawaz at nawazm3@cardiff.ac.uk

Supplementary Table 1. Regional assignments for all countries considered, as either a source or receptor, in this work.

Country Name	Region
Argentina	South America
Bolivia	South America
Brazil	South America
Chile	South America
Colombia	South America
Ecuador	South America
Guyana	South America
Peru	South America
Paraguay	South America
Suriname	South America
Uruguay	South America
Venezuela	South America
Australia	Oceania
Fiji	Oceania
Guam	Oceania
Kiribati	Oceania
New Zealand	Oceania
Papua New Guinea	Oceania
Solomon Islands	Oceania
Tonga	Oceania
Vanuatu	Oceania
Belize	North America
Canada	North America
Costa Rica	North America
Dominican Republic	North America
Guatemala	North America
Honduras	North America
Haiti	North America
Mexico	North America
Nicaragua	North America
Panama	North America
El Salvador	North America
United States of America	North America
Albania	Europe
Austria	Europe
Belgium	Europe
Bulgaria	Europe
Bosnia and Herzegovina	Europe
Belarus	Europe
Switzerland	Europe
Czechia	Europe

Germany	Europe
Denmark	Europe
Spain	Europe
Estonia	Europe
Finland	Europe
France	Europe
United Kingdom	Europe
Greece	Europe
Croatia	Europe
Hungary	Europe
Ireland	Europe
Italy	Europe
Lithuania	Europe
Luxembourg	Europe
Latvia	Europe
Republic of Moldova	Europe
North Macedonia	Europe
Malta	Europe
Netherlands	Europe
Norway	Europe
Poland	Europe
Portugal	Europe
Romania	Europe
Russian Federation	Europe
Serbia	Europe
Slovakia	Europe
Slovenia	Europe
Sweden	Europe
Ukraine	Europe
Iceland	Europe
Afghanistan	Asia
United Arab Emirates	Asia
Armenia	Asia
Azerbaijan	Asia
Bangladesh	Asia
Bahrain	Asia
Brunei Darussalam	Asia
Bhutan	Asia
China	Asia
Cyprus	Asia
Georgia	Asia
Indonesia	Asia
India	Asia
Iran	Asia
Iraq	Asia

Israel	Asia
Jordan	Asia
Kazakhstan	Asia
Kyrgyzstan	Asia
Cambodia	Asia
Republic of Korea	Asia
Kuwait	Asia
Lao Peoples Democratic Republic	Asia
Lebanon	Asia
Maldives	Asia
Myanmar	Asia
Mongolia	Asia
Malaysia	Asia
Nepal	Asia
Oman	Asia
Pakistan	Asia
Philippines	Asia
Democratic Peoples Republic of Korea	Asia
Qatar	Asia
Saudi Arabia	Asia
Singapore	Asia
Syrian Arab Republic	Asia
Thailand	Asia
Tajikistan	Asia
Turkmenistan	Asia
Turkey	Asia
Uzbekistan	Asia
Viet Nam	Asia
Yemen	Asia
Japan	Asia
Sri Lanka	Asia
Taiwan	Asia
Angola	Africa
Burundi	Africa
Benin	Africa
Burkina Faso	Africa
Botswana	Africa
Central African Republic	Africa
Côte dIvoire	Africa
Cameroon	Africa
Democratic Republic of the Congo	Africa
Congo	Africa
Djibouti	Africa
Algeria	Africa
Egypt	Africa

Eritrea	Africa
Ethiopia	Africa
Gabon	Africa
Ghana	Africa
Guinea	Africa
Gambia	Africa
Guinea-Bissau	Africa
Equatorial Guinea	Africa
Kenya	Africa
Liberia	Africa
Libya	Africa
Lesotho	Africa
Morocco	Africa
Mali	Africa
Mozambique	Africa
Mauritania	Africa
Malawi	Africa
Namibia	Africa
Niger	Africa
Nigeria	Africa
Rwanda	Africa
Sudan	Africa
Senegal	Africa
Sierra Leone	Africa
Somalia	Africa
Eswatini	Africa
Chad	Africa
Togo	Africa
Tunisia	Africa
United Republic of Tanzania	Africa
Uganda	Africa
South Africa	Africa
Zambia	Africa
Zimbabwe	Africa
Sao Tome and Principe	Africa
Mauritius	Africa
Madagascar	Africa
Comoros	Africa
Seychelles	Africa

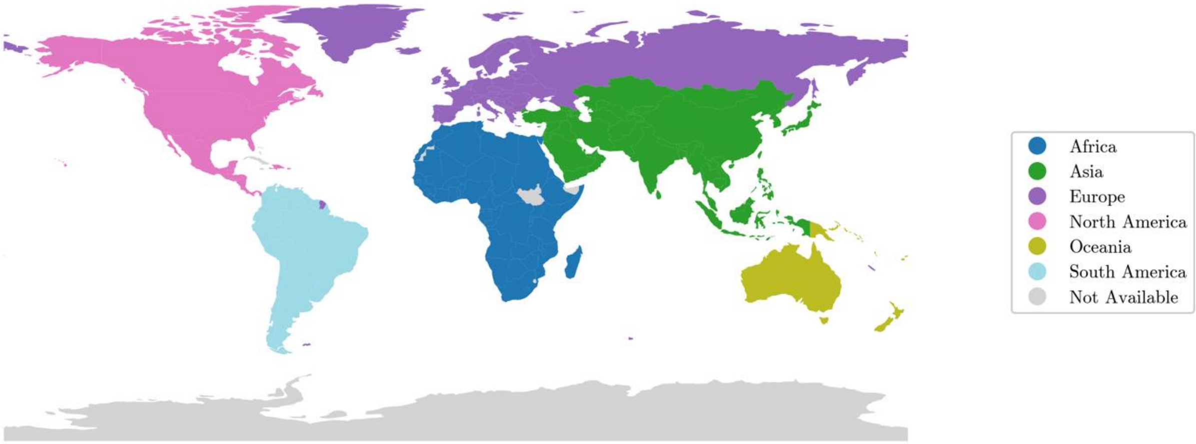

Supplementary Figure 1. Regional assignments for all countries considered, as either a source or receptor, in this work in map form.

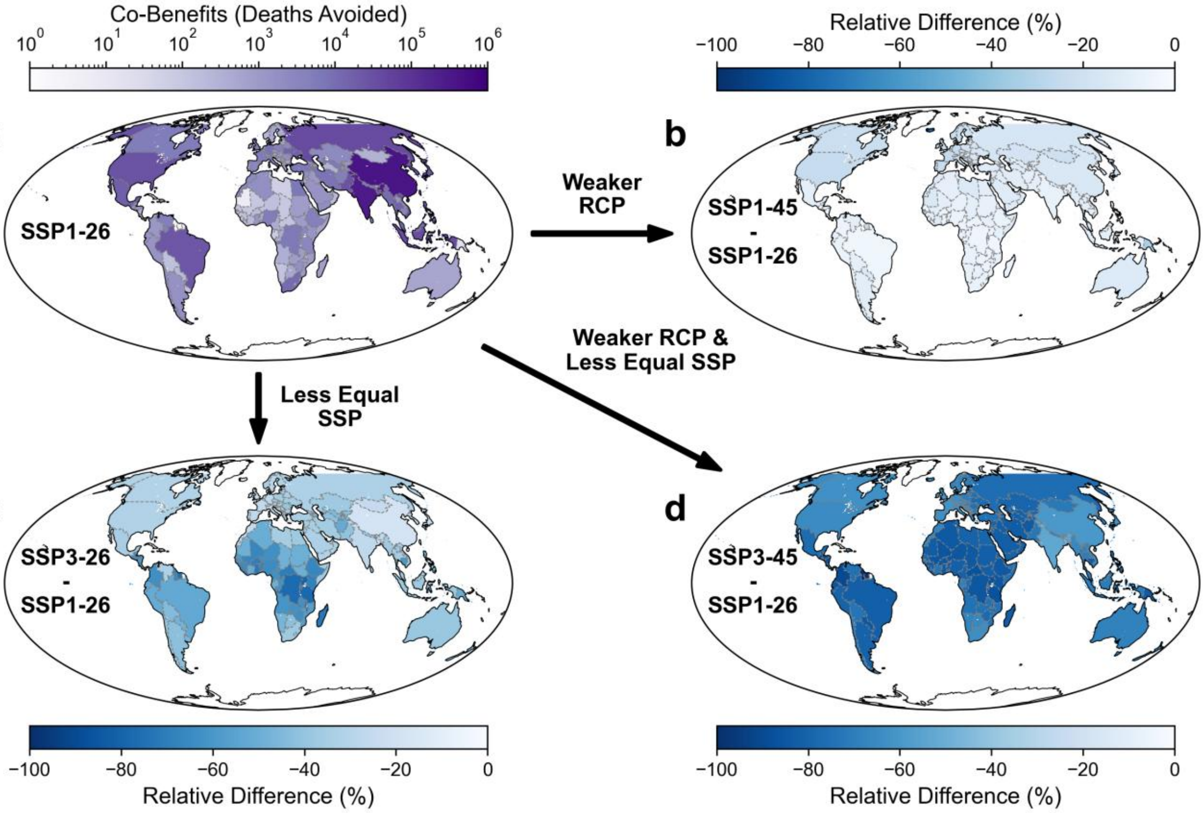

Supplementary Figure 2. The country-level total co-benefits in SSP1-26 (a) and the relatively fewer co-benefits in a weaker RCP (SSP1-45) (b), a less equal SSP (SSP3-26) (c), and both a weaker RCP and less equal SSP (SSP3-45) (d). The co-benefits in (a) are presented on a log-scale colormap; all other panels are presented on a linear colormap. Supplementary Figure 2 was created using Python and the Matplotlib, Cartopy, GeoPandas, and Contextily libraries. Country borders and coastlines are from Natural Earth (public domain), and the basemap is from CartoDB Positron (CC BY 4.0).

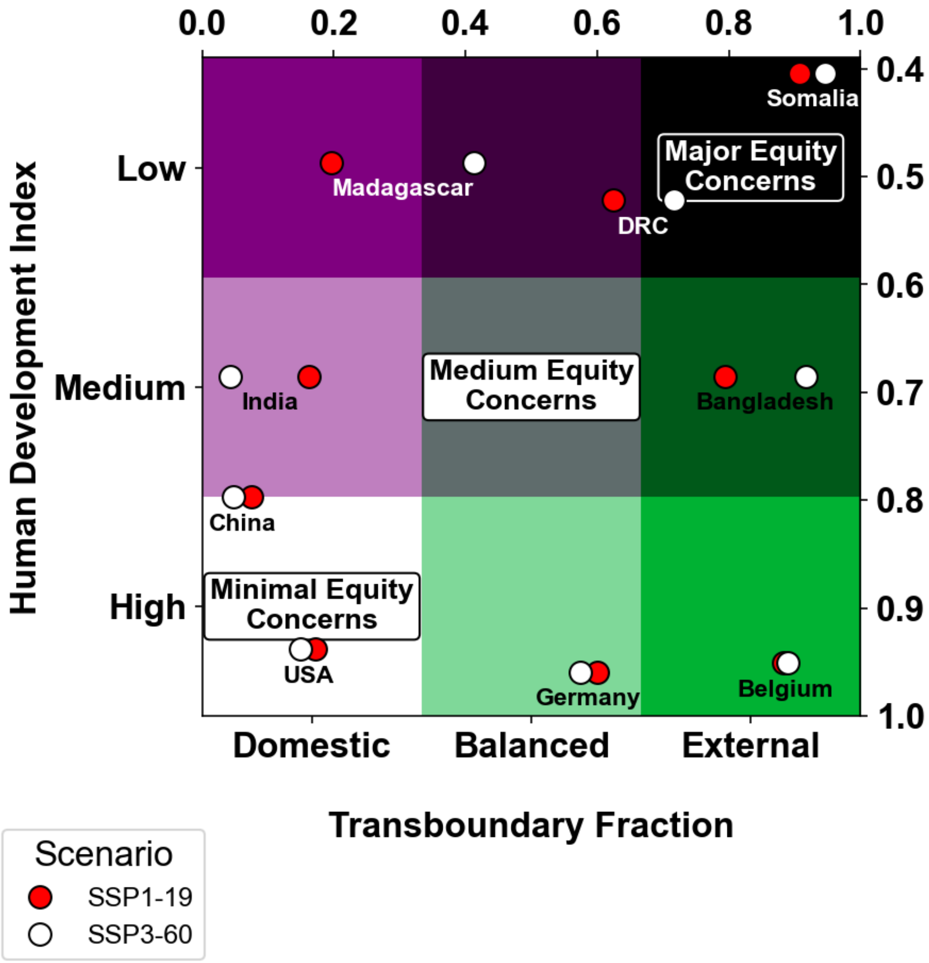

Supplementary Figure 3. Policy scorecard for determining whether a specific scenario had minimal, medium or major equity concerns based on the HDI value and transboundary fraction of the receptor country. Example values for SSP1-19 (Sustainability) and SSP3-60 (Fragmentation) are included for nine countries to indicate different values and how changing scenarios affect equity.

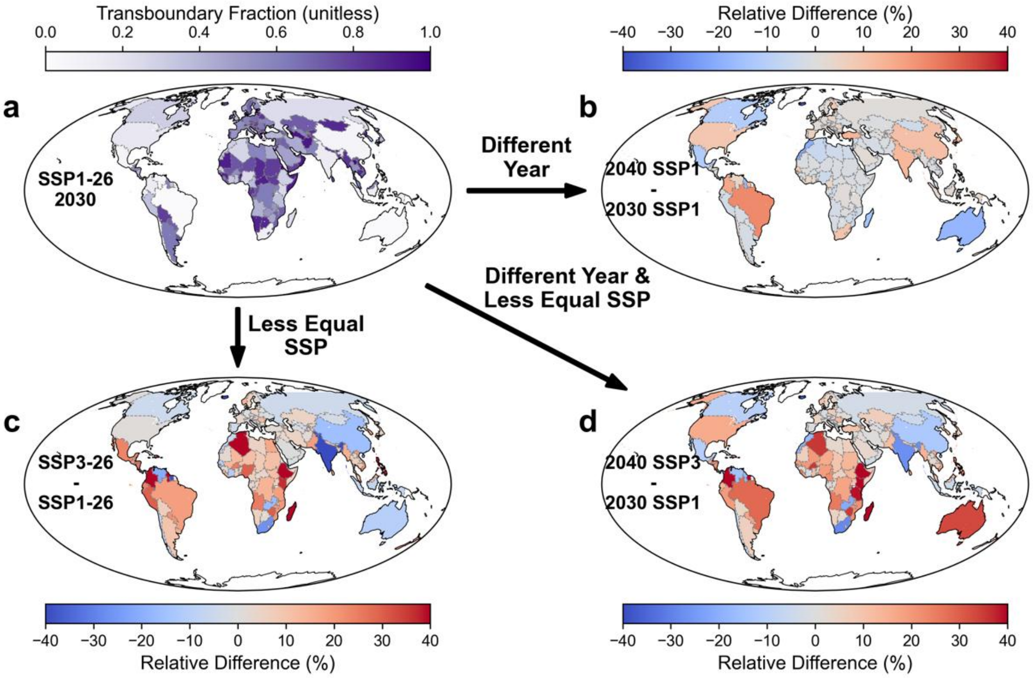

Supplementary Figure 4. Transboundary fractions for individual countries for a sustainable and strong climate forcing scenario (SSP1-26) in 2030 (a) and the percent difference in transboundary fractions for each country for the same scenario in 2040 (SSP1-26) (b), a less equitable SSP in 2030 (SSP3-26) (c) and the less equitable SSP in 2040 (d). Supplementary Figure 4 was created using Python and the Matplotlib, Cartopy, GeoPandas, and Contextily libraries. Country borders and coastlines are from Natural Earth (public domain), and the basemap is from CartoDB Positron (CC BY 4.0).

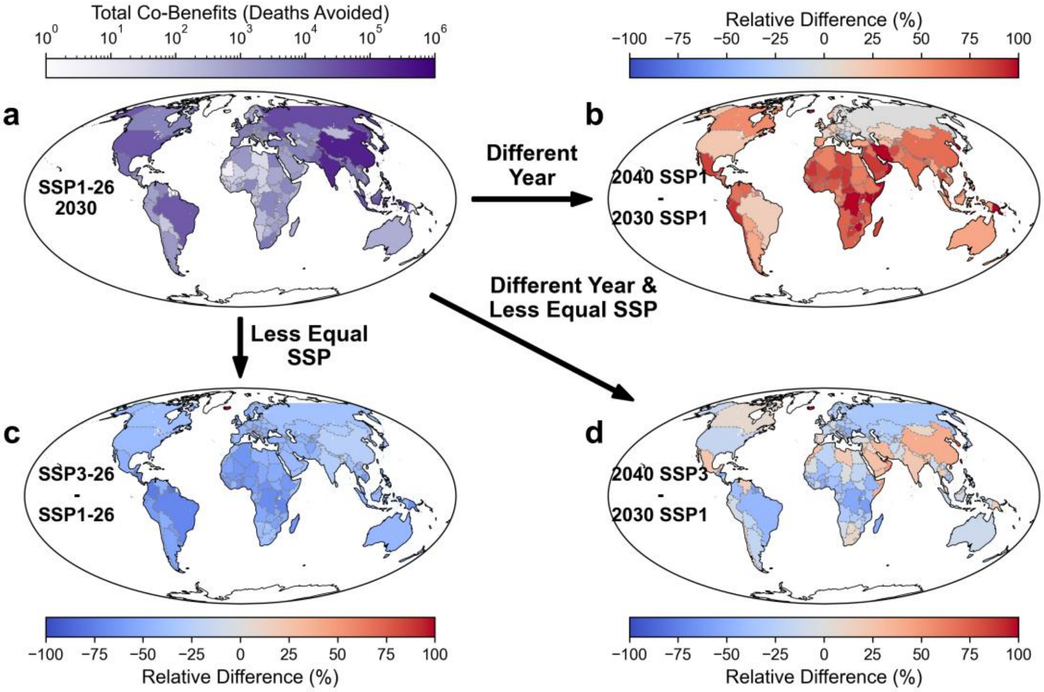

Supplementary Figure 5. Total co-benefits for individual countries for a sustainable and strong climate forcing scenario (SSP1-26) in 2030 (a) and the percent difference in transboundary fractions for each country for the same scenario in 2040 (SSP1-26) (b), a less equitable SSP in 2030 (SSP3-26) (c) and the less equitable SSP in 2040 (d). The total co-benefits in (a) are on a logarithmic color scale; all other panels are on linear color scales.

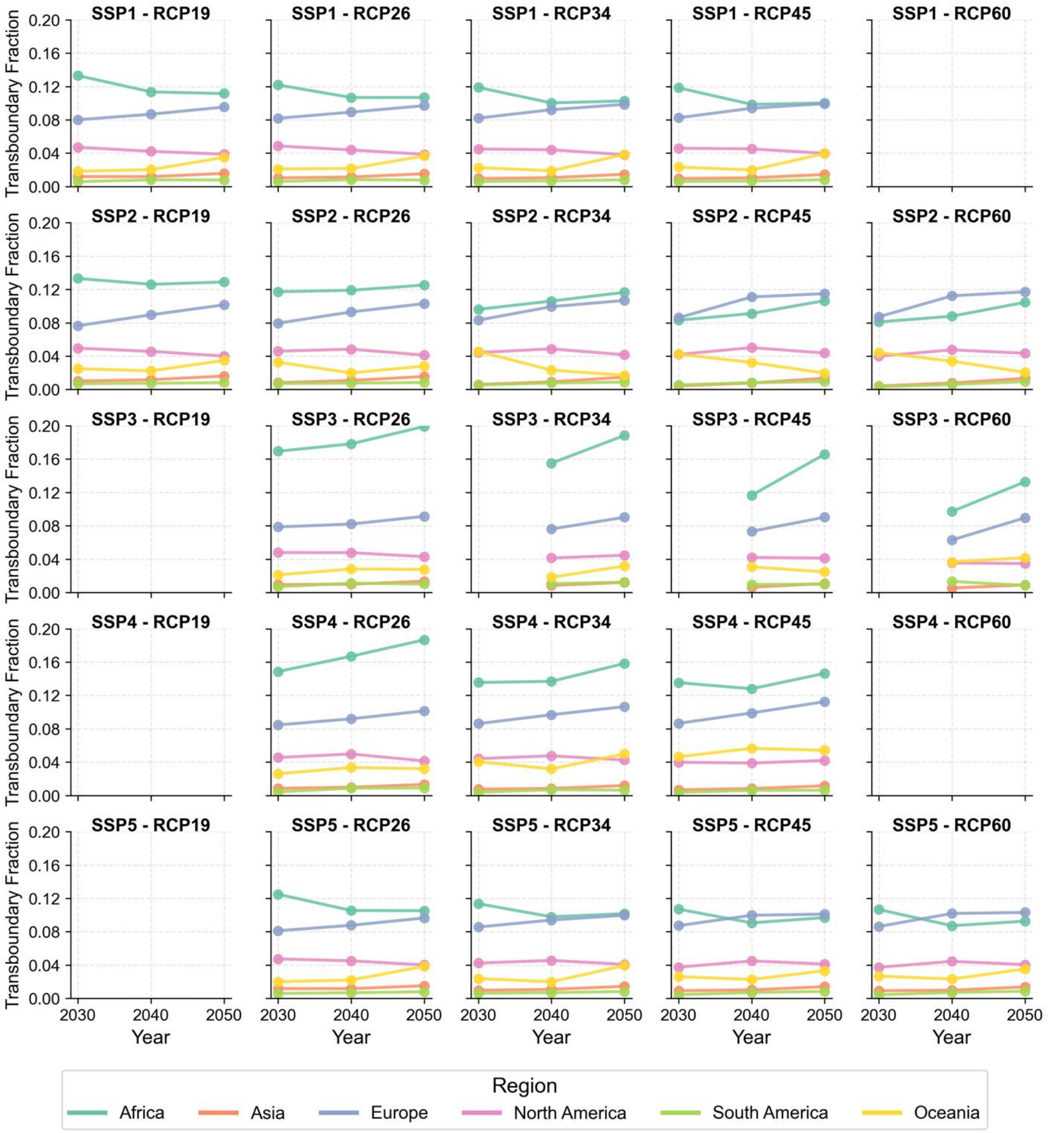

Supplementary Figure 6. Regional estimates of transboundary fractions for the six major regions for all scenarios considered in this study identified by rows (for SSPs) and

columns (for RCPs) across 2030, 2040, and 2050. For SSP3-34, SSP3-45, and SSP3-60, 2030 data are excluded as these scenarios were nearly identical to the baseline scenario. Supplementary Figure 6 was created using Python and the Matplotlib, Cartopy, GeoPandas, and Contextily libraries. Country borders and coastlines are from Natural Earth (public domain), and the basemap is from CartoDB Positron (CC BY 4.0).

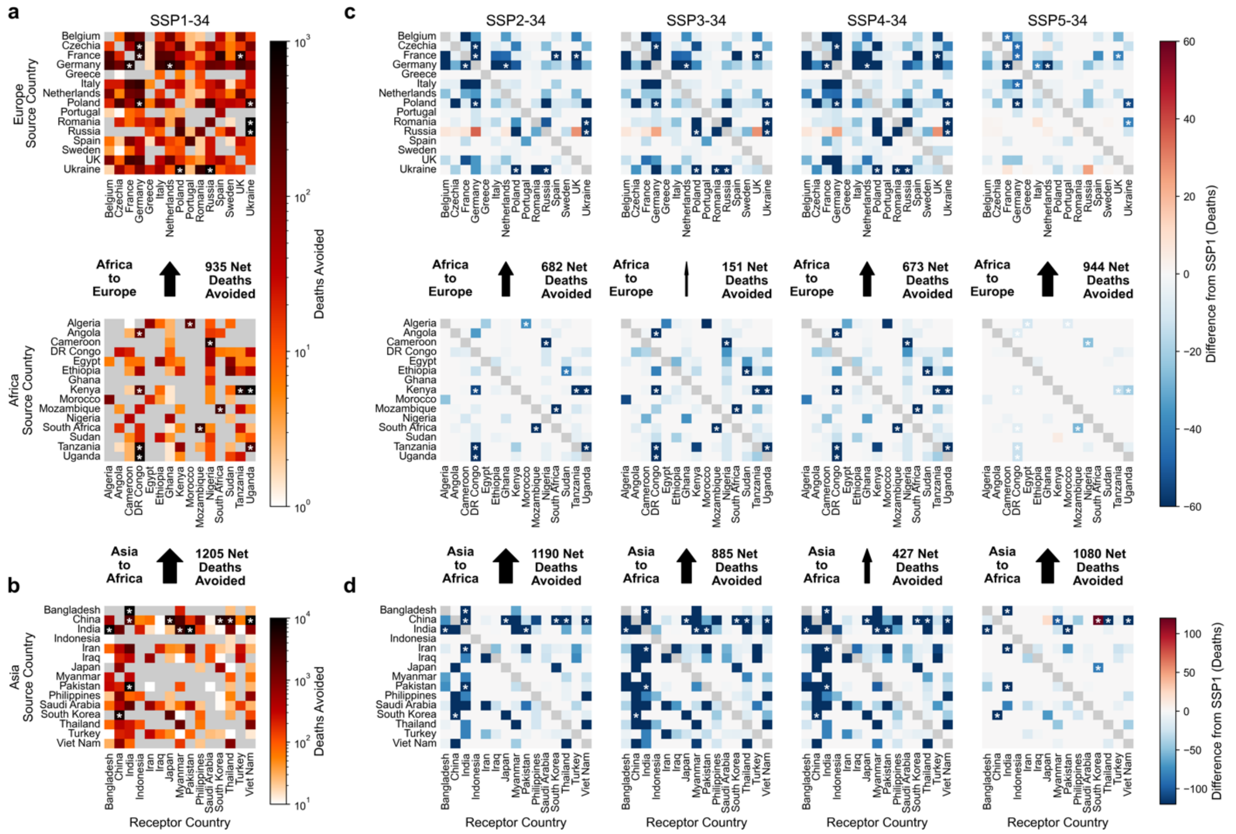

Supplementary Figure 7. Exchanges (EXC) of climate co-benefits within and between (a) Europe and Africa, for SSP1-34 in 2040 (left). Contributions are indicated through a logarithmic colormap ranging from 1 to 1000 deaths avoided. Darker colors indicate greater co-benefits in the receptor country attributable to emission reductions in the source country; self-contributions (i.e., the diagonal) are excluded. (b) EXC within Asia and between Asia and Africa; here contributions are indicated in a logarithmic colormap that ranges from 10 to 10000 deaths avoided. (c) Heatmaps of transboundary exchanges of climate action within and between Europe and Africa in 2040 for the scenarios SSP2-34, SSP3-34, SSP4-34, and SSP5-34 relative to SSP1-34. The colormaps are linear and range from -60 to +60 fewer or more deaths avoided compared to SSP1. (d) Heatmaps of transboundary exchanges of climate action within Asia and between Asia and Africa in 2040 for the scenarios SSP2-34, SSP3-34, SSP4-34, and SSP5-34 relative to SSP1-34. The colormaps are linear and range from -120 to +120 fewer or more deaths avoided compared to SSP1. For all subplots, interregional exchanges (i.e., Africa to Europe and Asia to Africa) are provided in an absolute sense – not relative to SSP1. White asterisks are placed to indicate the top 5% highest

(absolute) values for each heatmap. Same as Figure 4 in the main manuscript but for RCP-34 instead of RCP-26.

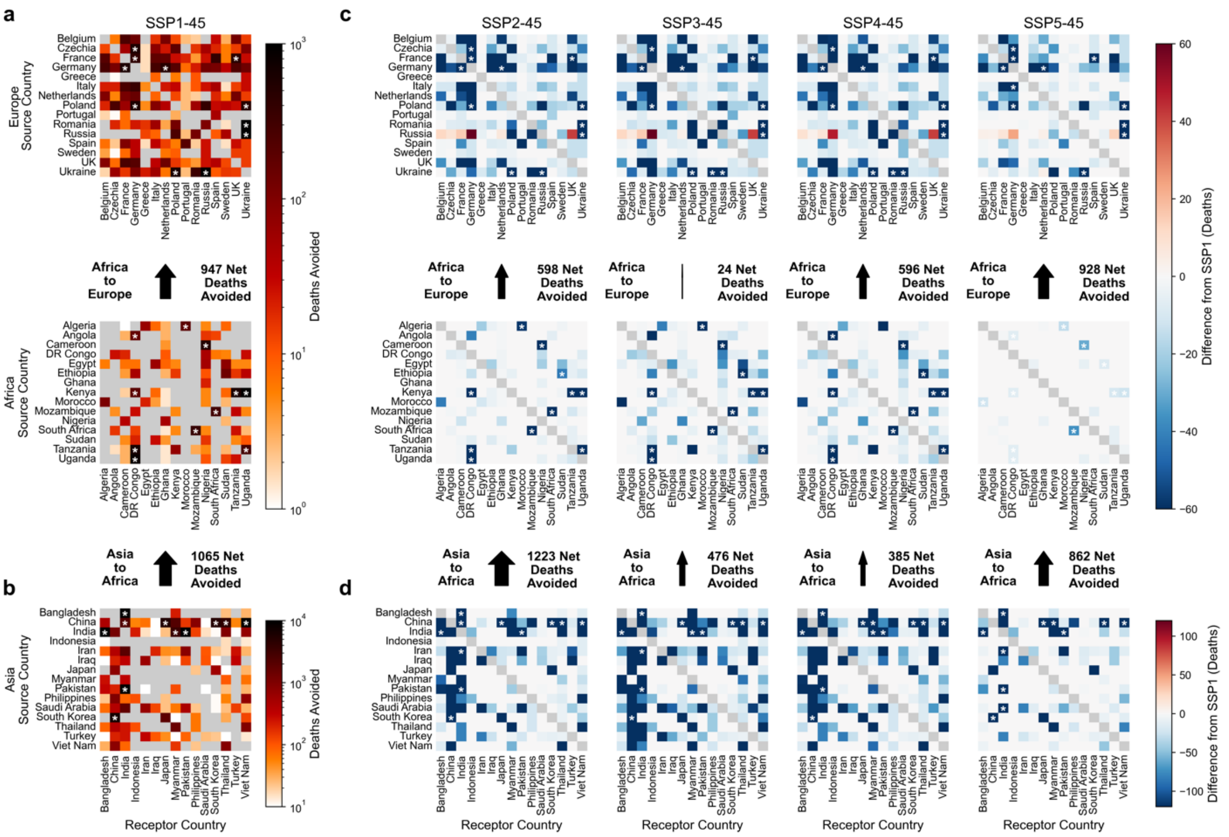

Supplementary Figure 8. Exchanges (EXC) of climate co-benefits within and between (a) Europe and Africa, for SSP1-45 in 2040 (left). Contributions are indicated through a logarithmic colormap ranging from 1 to 1000 deaths avoided. Darker colors indicate greater co-benefits in the receptor country attributable to emission reductions in the source country; self-contributions (i.e., the diagonal) are excluded. (b) EXC within Asia and between Asia and Africa; here contributions are indicated in a logarithmic colormap that ranges from 10 to 10000 deaths avoided. (c) Heatmaps of transboundary exchanges of climate action within and between Europe and Africa in 2040 for the scenarios SSP2-45, SSP3-45, SSP4-45, and SSP5-45 relative to SSP1-45. The colormaps are linear and range from -60 to +60 fewer or more deaths avoided compared to SSP1. (d) Heatmaps of transboundary exchanges of climate action within Asia and between Asia and Africa in 2040 for the scenarios SSP2-45, SSP3-45, SSP4-45, and SSP5-45 relative to SSP1-45. The colormaps are linear and range from -120 to +120 fewer or more deaths avoided compared to SSP1. For all subplots, interregional exchanges (i.e., Africa to Europe and Asia to Africa) are provided in an absolute sense – not relative to SSP1. White asterisks are placed to indicate the top 5% highest (absolute) values for each heatmap. Same as Figure 4 in the main manuscript but for RCP-45 instead of RCP-26.

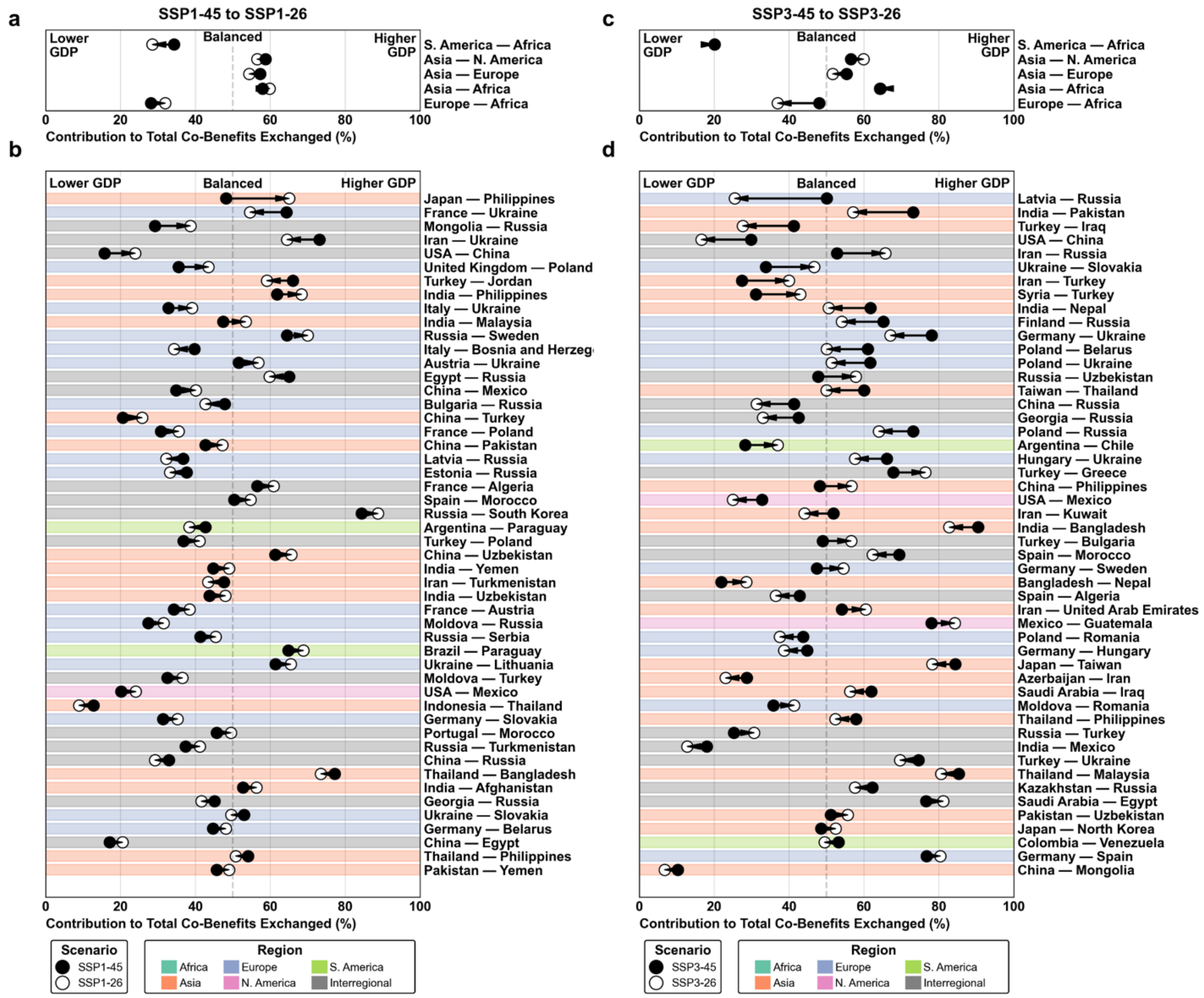

Supplementary Figure 9. The change in the percentage of co-benefits exchanged (TEC × 100%) between (a) regional and (b) country pairs that is contributed by the higher GDP (first name) from SSP1-45 to SSP1-26 in 2040. For the regional exchanges, the arrow points from the higher radiative forcing scenario (SSP1-45) to the lower radiative forcing scenario (SSP1-26) and the black dot represents SSP1-45 and the white dot represents SSP1-26. For the country exchanges, the arrow and dots are the same but the background shading indicates in which region the exchange occurs as labelled in the legend below the subplots; grey indicates exchange between different regions. Panels (c) and (d) are the same as (a) and (b), respectively, but for SSP3 – Fragmentation instead of SSP1.

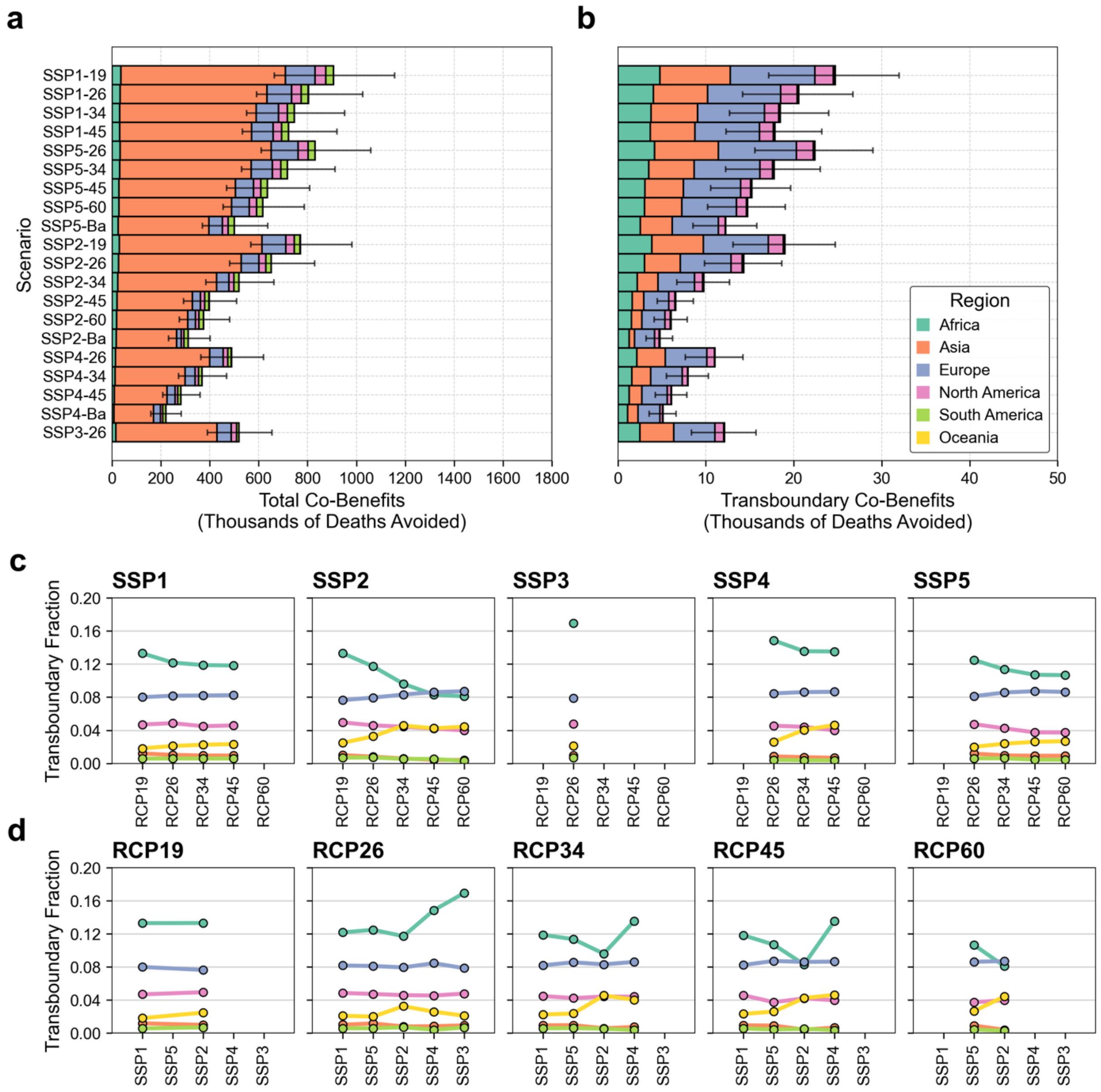

Supplementary Figure 10. (a) The total deaths avoided for each of the SSP-RCP scenarios relative to SSP3-Baseline in 2030 ordered from the most to least equitable scenarios. Colors indicate the receptor region in which the co-benefits occurred (i.e., where the deaths were avoided). Socioeconomic trends and mitigation strategies are included on the y-axis. Error bars refer to the lower and upper bound uncertainty from the health impact assessment for the total co-benefits. (b) The co-benefits specifically attributable to external action for each of the receptor regions (i.e., the transboundary co-benefits). The fraction of co-benefits that are transboundary broken down by (c) socioeconomic development type and (d) mitigation strategy. Same as Figure 2 in the main text but for 2030

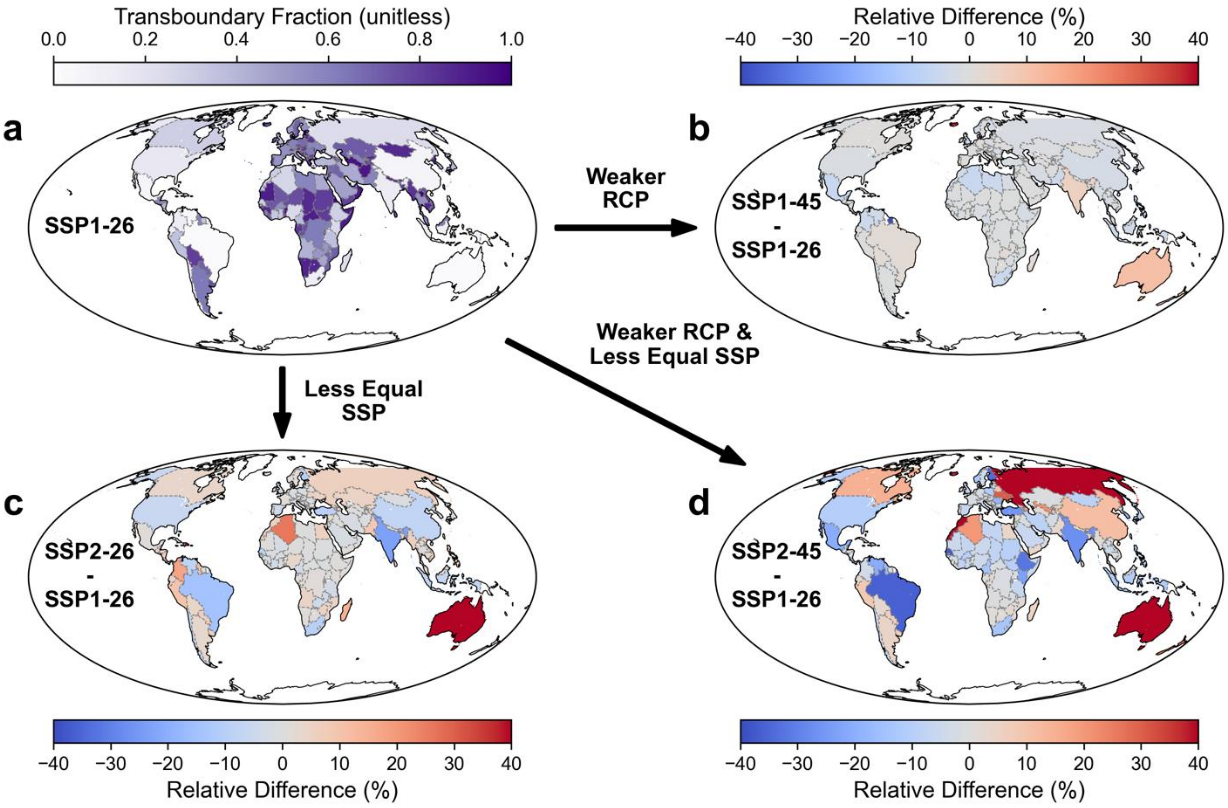

Supplementary Figure 11. (a) Transboundary fractions for individual countries in a sustainable and strong climate mitigation scenario (SSP1-26) in 2030, (b) the relative change in transboundary fractions associated with a weaker RCP, i.e., from SSP1-26 to SSP1-45; red values indicate where transboundary fractions have increased whereas blue values indicate decreases, (c) the relative change in transboundary fractions from a less equal SSP, i.e., from SSP1-26 to SSP2-26, (d) the relative change in transboundary fractions from both a weaker RCP and less equal SSP, i.e., from SSP1-26 to SSP2-45. Same as Figure 3 but for 2030 and the “less equal SSP” was replaced with SSP2 instead of SSP3 because SSP3-45 does not differ from the SSP3-Baseline in 2030. Supplementary Figure 11 was created using Python and the Matplotlib, Cartopy, GeoPandas, and Contextily libraries. Country borders and coastlines are from Natural Earth (public domain), and the basemap is from CartoDB Positron (CC BY 4.0).

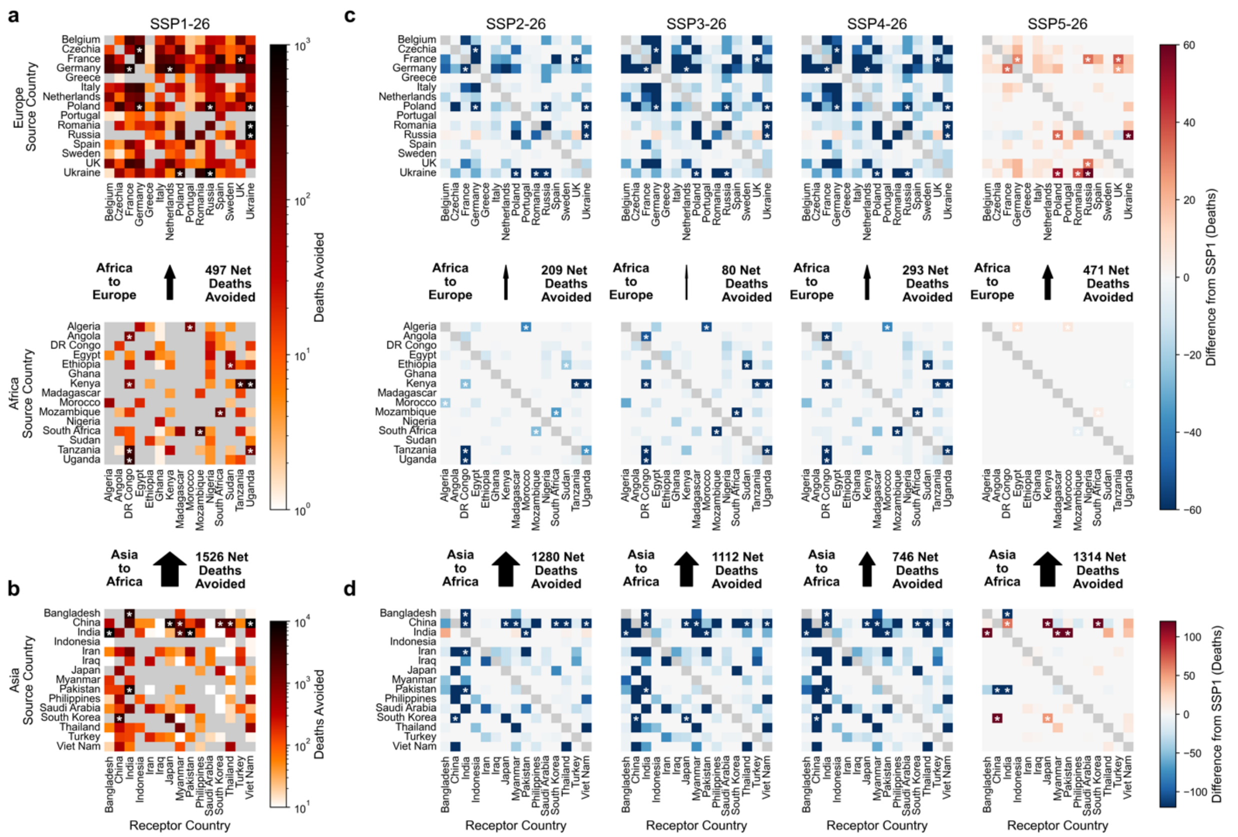

Supplementary Figure 12. Exchanges (EXC) of climate co-benefits within and between (a) Europe and Africa, for SSP1-26 in 2030 (left). Contributions are indicated through a logarithmic colormap ranging from 1 to 1000 deaths avoided. Darker colors indicate greater co-benefits in the receptor country attributable to emission reductions in the source country; self-contributions (i.e., the diagonal) are excluded. (b) EXC within Asia and between Asia and Africa; here contributions are indicated in a logarithmic colormap that ranges from 10 to 10000 deaths avoided. (c) Heatmaps of transboundary exchanges of climate action within and between Europe and Africa in 2030 for the scenarios SSP2-26, SSP3-26, SSP4-26, and SSP5-26 relative to SSP1-26. The colormaps are linear and range from -60 to +60 fewer or more deaths avoided compared to SSP1. (d) Heatmaps of transboundary exchanges of climate action within Asia and between Asia and Africa in 2040 for the scenarios SSP2-26, SSP3-26, SSP4-26, and SSP5-26 relative to SSP1-26. The colormaps are linear and range from -120 to +120 fewer or more deaths avoided compared to SSP1. For all subplots, interregional exchanges (i.e., Africa to Europe and Asia to Africa) are provided in an absolute sense – not relative to SSP1. White asterisks are placed to indicate the top 5% highest (absolute) values for each heatmap. Same as Figure 4 in the main text but for 2030.

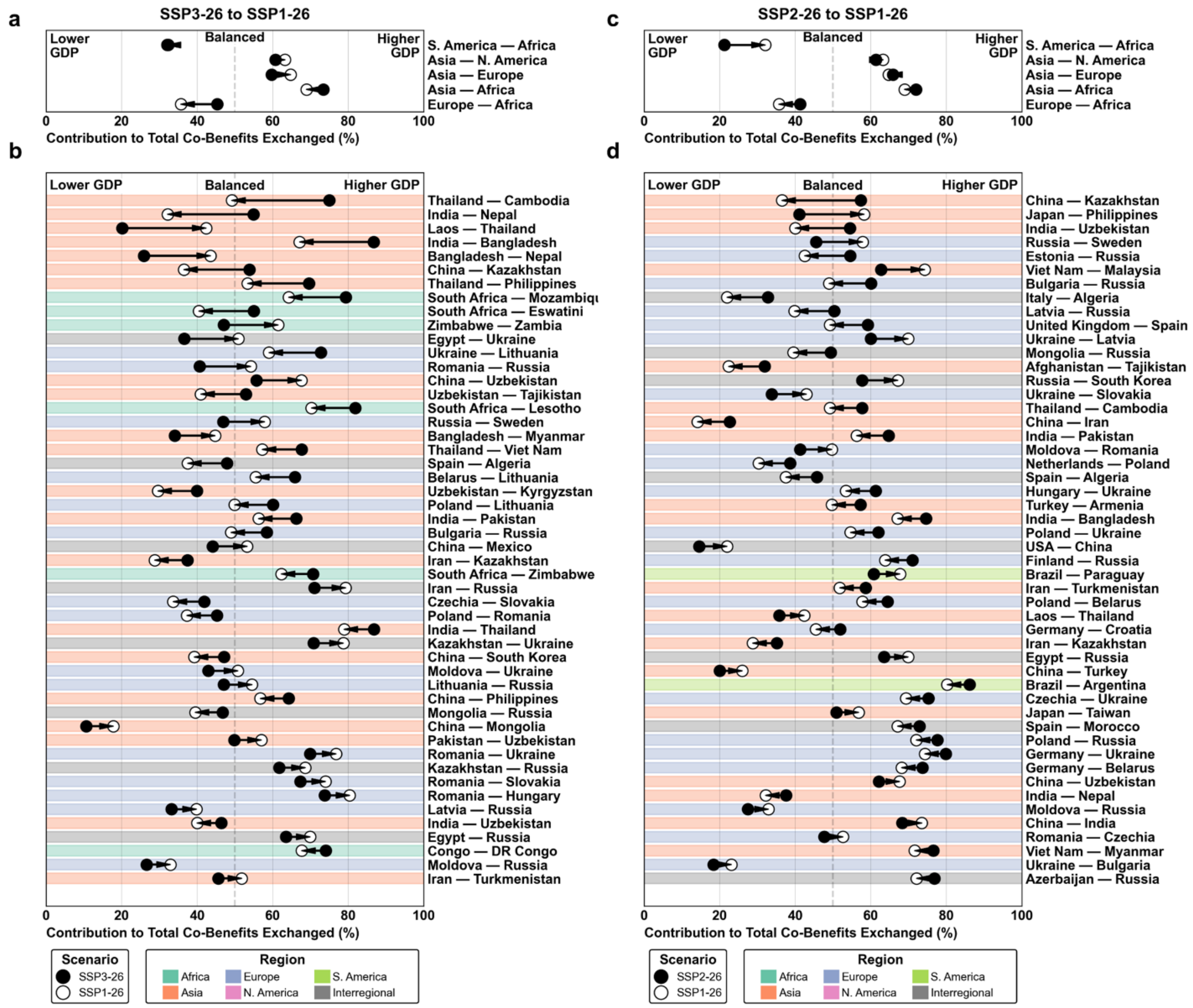

Supplementary Figure 13. The change in the percentage of co-benefits exchanged between regional (a) and country (b) pairs that is contributed by the higher GDP (first name) from SSP3-26 to SSP1-26 in 2030. This figure is in the same style as Figure 5 in the main text but for 2030; however, we RCP-26 instead of RCP-45 given that in 2030, the RCP-45 scenario is the same as the SSP3-Baseline. For the regional exchanges, the arrow points from the less equal scenario (SSP3-26) to the more equal scenario (SSP1-26) and the color of the dot indicates the higher regional contributor. For the country exchanges, the arrow is the same but the black dot represents SSP3-26 and the white dot represents SSP1-26 and the background shading indicates in which region the exchange occurs as labelled above Figure S9a; grey indicates exchange between different regions. The change in the percentage of co-benefits exchanged between regional (c) and country (d) pairs that is contributed by the higher GDP (first name) from SSP2-26 to SSP1-26; this is the same as Figure S9a and S9b except it explores middle-of-the-road development instead of fragmentation development.
